# Preoperative Surgical Fear, Postoperative Pain, and Sleep Quality in Metabolic and Bariatric Surgery Patients: A Descriptive and Correlational Study

**DOI:** 10.1007/s11695-025-07770-3

**Published:** 2025-03-04

**Authors:** Sevgi Deniz Doğan, Dilek Güneş, Şeyma Yurtseven, Sevban Arslan, Muaz Gülşen, Cüneyt Kirkil

**Affiliations:** 1 Isparta University of Applied Sciences, Isparta, Turkey; 2 Fırat University, Elâzığ, Turkey; 3 Cukurova University, Adana, Turkey

**Keywords:** Bariatric surgery, Fear, Nursing, Postoperative pain, Sleep quality

## Abstract

**Background:**

This study aimed to determine the preoperative surgical fear levels, postoperative pain intensity, and sleep quality of metabolic and bariatric surgery patients and to examine the relationship between them.

**Methods:**

This study was a single-center descriptive and correlational design and was completed with 115 patients. The data of the study were collected by using the Personal Information Form, Surgical Fear Questionnaire, Richards-Campbell Sleep Questionnaire, and Visual Analog Scale. Descriptive statistics, Pearson correlation, and multiple linear regression analysis were used for data analysis.

**Results:**

In the study, the mean total score of the preoperative Surgical Fear Questionnaire was 24.73 ± 16.32, and the mean total score of the Richards-Campbell Sleep Questionnaire on the 1st postoperative day was 53.85 ± 20.53. The mean postoperative pain intensity scores were 7.44 ± 1.74 at the 1st hour, 5.19 ± 1.61 at the 12th hour, and 3.24 ± 1.85 at the 24th hour. In addition, it was determined that surgical fear and postoperative pain significantly predicted sleep quality (*p* < 0.05).

**Conclusions:**

It was determined in the study that sleep quality decreased as the level of surgical fear and pain intensity of the patients increased. Considering the relationship between fear of surgery, postoperative pain, and sleep quality, it may be beneficial to develop support and counseling services for patients according to current guidelines.

## Introduction

According to the definition of the World Health Organisation (WHO), obesity is a chronic, complex disease characterized by excessive accumulation of fat in the body to the extent that it can harm health [1]. In a simpler definition, obesity is defined as a BMI higher than 30 kg/m^2^ [2]. Obesity is a serious health problem that is widespread all over the world. According to 2022 WHO data, more than 2.5 billion adults worldwide are overweight and 890 million are obese [1].

Obesity, which has become a global public health problem today, negatively affects the health of many individuals [3]. There are various methods for treating obesity, such as medical nutrition therapy, exercise, behavior therapy, and medication [4]. However, metabolic and bariatric surgery (MBS) is currently the most effective treatment for the control of severe obesity and associated metabolic disorders [5].

According to the 2022 update by the American Society for Metabolic and Bariatric Surgery (ASMBS) and International Federation for the Surgery of Obesity and Metabolic Disorders (IFSO), MBS is recommended for individuals with BMI ≥ 35 kg/m^2^ regardless of the presence, absence, or severity of comorbidities. It is also reported that MBS should be considered for individuals with metabolic disease and BMI of 30–34.9 kg/m^2^ [2].

MBS, which is the most effective method in the treatment of severe obesity, is becoming safer and safer [6]. However, like all surgical procedures, MBS affects patients in many physiological and psychological aspects. Surgical fear, which starts in patients after the surgical intervention decision is taken, is among the most common psychological problems experienced by patients in the preoperative period [7–9]. This fear experienced by patients may affect the perception and severity of postoperative pain [10, 11]. Patients may feel more pain in the postoperative period due to the high level of fear they experience before surgery [12, 13]. In addition, surgical fear may also negatively affect sleep quality in the postoperative period [12]. Anxiety and fear may impair sleep quality and negatively affect the recovery process of patients. A good quality of sleep after surgery plays a critical role in accelerating the recovery process and improving overall health [14].

Nowadays, MBS is commonly performed with a laparoscopic approach and patients experience less postoperative pain [15]. However, patients undergoing bariatric surgery still experience pain of varying intensity [16]. The pain experienced by patients after surgery can directly affect their sleep quality [12]. Poor sleep quality can also negatively affect the healing process and increase the perception of pain [17]. Therefore, understanding the relationship between surgical fear, postoperative pain, and sleep quality experienced by patients before MBS may help to optimize the recovery process. It may also enable the development of more effective treatment and support programs before and after surgery.

## Methods

### Aims

This study aimed to determine the preoperative surgical fear levels, postoperative pain intensity, and sleep quality of MBS patients and to examine the relationship between them.

### Study Design

This study is a single-center descriptive and correlational design. This study was structured and reported according to the Strengthening the Reporting of Observational Studies in Epidemiology (STROBE) checklist [18].

### Setting and Participants

The population of the study consisted of patients who were hospitalized in the general surgery clinic of a university hospital, met the research criteria, and were planned to undergo sleeve gastrectomy or gastric bypass operation by laparoscopic method. The inclusion criteria were to be over 18 years of age, to be literate, to have no speech and hearing impairment, to undergo general anesthesia, to undergo MBS for the first time, to be treated in the clinic for at least 24 h after surgical intervention, and to agree to participate in the study. Exclusion criteria were postponement or cancellation of the surgery, development of postoperative complications or intensive care unit admission, and the patient’s desire to leave the study. The sampling required for the study was calculated using the G*Power 3.1 software statistical analysis software based on a 0.05 significance level (*α* err prob), 95% power (1-*β* = 0.95), and 0.15 effect size (*f*^2^) as medium effect size. As a result, the sample size required for multiple linear regression analysis was determined to be at least 89 patients [19, 20]. The study was completed with 115 patients. As a result of the post-hoc power analysis, the power of the study was calculated as 99% with a large effect size (1.17) and a margin of error of 0.05.

### Instruments

The data of the study were collected by using the Personal Information Form, Surgical Fear Questionnaire (SFQ), Richards-Campbell Sleep Questionnaire (RCSQ), and Visual Analog Scale (VAS).

The personal information form consisted of questions such as age, gender, marital status, educational status, employment status, previous surgery experience, presence of chronic disease, regular medication use, smoking, and alcohol use.

The SFQ consists of eight items. The scores obtained from the items range from 0 to 10 (0 “not at all afraid,” 10 “very afraid”). The lowest score that can be obtained from the scale is 0, while the highest score is 80. The first 4 items (SFQ-S) in the scale measure the fear of short-term consequences of surgery and the last 4 items (SFQ-I) measure the fear of long-term consequences of surgery. Bağdigen and Karaman Özlü (2016) found the Cronbach alpha coefficient of the scale to be 0.89 [21].

VAS consists of a 10-cm long line drawn vertically or horizontally. On a 10-cm ruler, where there is no pain at one end and the most severe pain at the other end, the patient marks his or her pain. The length of the distance from the point where there is no pain to the point marked by the patient indicates the patient’s pain [22].

RCSQ consists of 6 items and each item is evaluated on a scale from 0 to 100. As the score obtained from the scale increases, the sleep quality of the patients is interpreted as increasing. In addition, a score between “0 and 25” indicates very poor sleep quality, and a score between “76 and 100” indicates very good sleep quality. Karaman Özlü et al. (2015) found the Cronbach *α* reliability coefficient of the scale to be 0.91 [23].

### Data Collection

The data of the study were collected between November 2023 and May 2024. Patients who were planned for MBS who were treated in the general surgery clinic, who met the sampling criteria and who agreed to participate in the study were informed about the purpose of the study on the morning of surgery and their consent was obtained. Then, the personal information form, SFQ, and VAS were filled out. The pain of the patient who came to the clinic after the operation was evaluated at the 1st, 12th, and 24th hours using VAS. Sleep quality was also evaluated with RCSQ at the 24th hour. The patients were then thanked for their participation in the study.

### Ethical Considerations

To conduct the research; approval from the ethics committee of a university (Decision no: 166/02 Date: 25/10/2023) and necessary institutional permissions were obtained from the hospital where the research was conducted. The patients included in the study were informed about the research, the purpose of the research was explained and their verbal consent was obtained that they agreed to participate in the research. Patients were informed that their participation or withdrawal from the study would not affect their treatment and care. Identifying information such as name and surname of the participants were not asked in the data collection forms and all data were collected anonymously. The study was conducted in accordance with the principles of the Declaration of Helsinki.

### Data Analysis

Data were analyzed using SPSS 22.0 (IBM, Armonk, NY, USA). There was no missing data. The skewness and kurtosis values of the data were checked for the assumption of normality, and the values were found to range between − 2.0 and + 2.0 [24]. The data were evaluated with descriptive statistics. Mean, standard deviation, number, and percentage values were used in descriptive statistics.

Pearson correlation coefficient was calculated to determine the relationship between surgical fear, pain intensity, and sleep quality. Multiple linear regression analysis was used to test the relationship between independent and dependent variables. The normality assumption was assessed by plotting the quantiles of the model residuals against the quantiles of a Q-Q scatterplot and the plots indicate the data showed an approximately normal distribution [25]. Homoscedasticity was evaluated by plotting the residuals against the predicted values [26]. There were no multiple connection problems according to tolerance and Variance inflation factors (VIF) values (Tolerance > 0.1; VIF < 10). There was no autocorrelation among the independent variables (1.5 < Durbin-Watson value > 2.5) [27]. The categorical independent variables were coded dummy. *R*^2^ statistic assesses how well the regression predicted the dependent variable. While the unstandardized beta (*B*) describes the increase or decrease of the independent variable(s) with the dependent variable. For all analyses, the level of statistical significance was specified at *p* < 0.05.

### Validity and Reliability/Rigor

The STROBE checklist was used to report this study [18]. The measurement tools used in the study were validated for the Turkish population and showed good validity and reliability. Cronbach’s alpha coefficient was used to test the reliability of the scale items used in the current study. In this study, Cronbach’s alpha coefficient of SFQ and RCSQ was 0.90 and 0.91, respectively. The results showed that the measurement tools used in this study were reliable [28].

## Results

The mean age of the patients who participated in the study was 38.1 ± 12.43 years, 58.3% were female, 43.5% had secondary education, 55.7% were employed, and 66.1% were married. 33.9% of the patients had at least one chronic disease, 34.8% used regular medication, 45.2% smoked, and 9.6% used alcohol. 56.5% of the patients had previous surgical experience.

The mean scores of the patients from RCSQ, SFQ, and their sub-dimensions are given in Table 1. In the study, the mean score of the patients from the SFQ-S sub-dimension was 15.41 ± 10.08, the mean score from the SFQ-I sub-dimension was 9.32 ± 8.37 and the mean total score from the scale was 24.73 ± 16.32. The mean total score of the patients on the RCSQ was 53.85 ± 20.53 (Table 1).

**Table 1:** Distribution of the mean scores of the patients on the SFQ and RCSQ (*n* = 115)

	$\overline{\text{X}}\pm \text{SD }$	Median [min–max]
SFQ-S	15.41 ± 10.08	12.0 [0–39]
SFQ-I	9.32 ± 8.37	06.0 [0–33]
SFQ-T	24.73 ± 16.32	20.0 [0–69]
RCSQ	53.85 ± 20.53	59.0 [2–83]

**Fig. 1:**
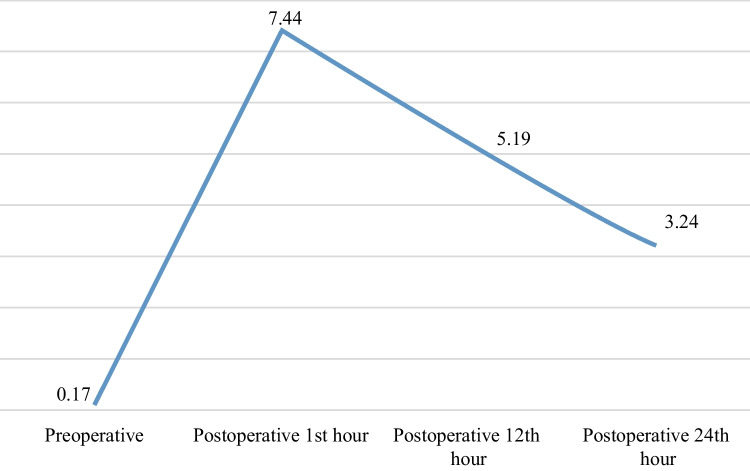
Pain intensity of the patients from preoperative to postoperative 24th hour (*n* = 115)

The change in the mean pain intensity scores of the patients according to time is shown in Fig. 1. The mean preoperative pain intensity score of the patients was 0.17 ± 0.80, 7.44 ± 1.74 at 1st hour, 5.19 ± 1.61 at 12th hours and 3.24 ± 1.85 at 24th hours postoperatively. The mean of three postoperative measurements was 5.29 ± 1.39.

A positive linear relationship was determined between preoperative SFQ-S and SFQ total scale mean scores and postoperative pain (*p* < 0.05). In this direction, as the mean SFQ score increases, the mean postoperative pain score also increases. In addition, a negative relationship was determined between RCSQ and postoperative pain, SFQ total score, and its sub-dimensions (*p* < 0.05). This means that as the mean scores of postoperative pain, SFQ total score, and sub-dimensions increase, the mean score of RCSQ decreases (Table 2).

**Table 2:** The relationship between preoperative surgical fear, postoperative pain, and sleep quality (*n* = 115)

		SFQ-S	SFQ-I	SFQ-T	Postoperative pain^1^	RCSQ
SFQ-S	*r* *p*	1.000	0.561** **0.000**	0.905** **0.000**	0.219* **0.019**	− 0.683** **0.000**
SFQ-I	*r* *p*		1.000	0.859** **0.000**	0.139 0.137	− 0.607** **0.000**
SFQ-T	*r* *p*			1.000	0.207* **0.027**	− 0.733** **0.000**
Postoperative pain^1^	*r* *p*				1.000	− 0.282* **0.002**
RCSQ	*r* *p*					1.000

*α significance level is taken as 0.05, *r* Pearson correlation, *p* test statistic value, 1 mean of three postoperative measurements (1st, 12th, and 24th hour)

The results of the model established in the linear regression analysis were significant (F = 17.704, *p* < 0.001, *R*^2^ = 0.572, Adj. *R*^2^ = 0.540) and showed that approximately 54% of the variance of the model could be explained (Table 3). It was determined that surgical fear and postoperative pain significantly predicted sleep quality (*p* < 0.05). The study determined that a one-unit increase in the patients’ average score on the SFQ-S would reduce the average RCSQ score by 0.465 units. Additionally, it was determined that a one-unit increase in the patients’ average score on the SFQ-I would reduce the average RCSQ score by 0.330 units.

**Table 3:** Factors affecting sleep quality after bariatric surgery with regression analysis (*n* = 115)

Independent variable	*B*	*SE*	*β*	*t*	*p*	95% CI		Tolerance	VIF
						Lower	Upper		
Constant	78.142	7.258		10.766	.000	63.752	92.531		
Gender (reference value = male)									
Female	1.865	2.771	0.045	0.691	0.491	− 3.490	7.221	0.950	1.052
Chronic disease (reference value = no)									
Yes	0.480	3.103	0.011	0.155	0.877	− 5.672	6.631	0.781	1.280
Smoking (reference value = no)									
Yes	2.324	2.784	− 0.094	0.834	0.406	− 3.201	7.848	0.876	1.141
Alcohol (reference value = no)									
Yes	− 1.258	4.842	− 0.018	− 0.260	0.796	− 10.858	8.342	0.831	1.203
Age	0.159	0.114	0.096	1.388	0.168	− 0.068	0.385	0.842	1.188
Postoperative pain^1^	− 0.196	0.098	− 0.133	1.990	**0.049**	− 0.390	− 0.001	0.905	1.106
SFQ-S	− 0.948	0.164	− 0.465	5.784	**0.000**	− 1.272	− 0.623	0.624	1.604
SFQ-I	− 0.809	0.192	− 0.330	− 4.210	**0.000**	− 1.190	− 0.428	0.657	1.522

*R* = 0.756; *R*^2^ = 0.572; Adjusted *R*^2^ = 0.540

*F* = 17.704, *p* < 0.001

Durbin-Watson = 1.629* *α* significance level is taken as 0.05,B: standardized regression coefficient, *SE* standard error; *95% CI*, 95% confidence intervals, *p* test statistic value, 1: mean of three postoperative measurements (1st, 12th, and 24th hour)

The findings show that a one-unit increase in patients’ average postoperative pain will reduce the average sleep quality score by 0.133 units. However, gender, age, chronic disease, smoking, and alcohol use were not seen as a predictor of sleep quality in the errors (*p* > 0.05).

## Discussion

MBS, which is recognized as the most effective method in the treatment of severe obesity, is becoming more and more common today [5]. Therefore, investigating MBS in all aspects will be effective in improving the quality of care. In the study, preoperative surgical fear levels, postoperative pain intensity and sleep quality on the first postoperative day were evaluated and a significant relationship was found between them. In this section, the findings of the study are discussed with the literature.

It can be said that the preoperative surgical fear level of the patients in the study was low (24.73 ± 16.32), although the scale used did not have a cut-off point. In the survey conducted by Odabaşi et al. [12], it is seen that the fear level of patients before bariatric surgery is close to this study [12]. In studies conducted in the literature with patients undergoing different elective surgical procedures, it is noteworthy that the level of surgical fear is relatively higher compared to MBS patients [8, 9, 29]. These results may be related to the fact that MBS is becoming more widespread day by day, success rates are known, and patients are more informed about the safety and effectiveness of such surgeries. In addition, in the study, it was observed that patients experienced more fear of the short-term consequences of surgery according to their mean scores on the scale. Similar results were found in the literature [12, 30]. This may be because the short-term results of surgery usually involve direct and concrete experiences such as pain and complications.

In the study, it was determined that the pain intensity of the patients was severe (7.44 ± 1.74) in the first postoperative measurement and tended to decrease gradually in the first 24 h. Similar results are also found in the literature [16, 31, 32]. In a study, it was reported that nearly 75% of bariatric surgery patients experienced moderate to severe pain in the first 24 h after surgery [32]. In the study by Gravani et al. [12], it was reported that the severe and uncomfortable pain of patients after bariatric surgery at the 1st hour decreased to moderate intensity 4 h after surgery and continued until the 8th hour after surgery [16]. These findings suggest that more effective strategies for pain control in the early postoperative period after MBC should be developed.

Poor sleep quality after surgery may affect the recovery process and increase the risk of complications [14]. A study reported that poor sleep quality is common (64.9%) after surgery [33]. In this study, when the sleep quality of patients was evaluated on the first postoperative day, it was determined that their sleep quality was moderate according to the cut-off point of the scale. In the literature, studies evaluating the sleep quality of MBS patients in the early postoperative period are very limited. In one study, the sleep quality evaluated by bariatric surgery patients at hospital discharge was found to be moderate [12]. These findings may be the result of the persistence of problems such as pain, anesthesia effects, hospital stay discomfort, and sleep apnoea in the early postoperative period.

In the study, it was found that there was a relationship between the preoperative surgical fear level of the patients and postoperative sleep quality and that a one-unit increase in patients’ fear of the short-term consequences of surgery decreased sleep quality by approximately half afold. In addition, it was found that a one-unit increase in fear of the long-term consequences of surgery decreased sleep quality by 0.33 times. Odabaşi et al. [12] also reported that increase in patients’ fear of short-term consequences of surgery decreased sleep quality. However, the researchers did not report a relationship between patients’ fears about the long-term consequences of surgery and sleep quality in the study [12]. A study in the literature with different procedures also showed that fear of surgery was associated with postoperative sleep quality [34]. These findings suggest that the fear experienced by patients in the preoperative period may affect the recovery process and sleep quality in the postoperative period. Therefore, it is important to consider and appropriately manage patients’ fears in the preoperative period.

In the study, it was found that there was a relationship between the postoperative first-day pain mean score and sleep quality, and a one-unit increase in pain decreased sleep quality by 0.13 times. Similar to this study, no study examining the relationship between pain after MBC and sleep quality was found in the literature. However, studies conducted with different surgical procedures are consistent with this study [33, 35]. Tegegne and Alemnew [33] reported a relationship between poor postoperative sleep quality and moderate and severe pain history in a multicentre study [33]. These findings emphasize that pain and sleep problems experienced by patients in the postoperative period should be addressed together. Therefore, it is important not only to reduce pain but also to improve sleep quality in pre-and postoperative pain management plans.

### Limitations and strengths

This study has several limitations. First, since it is a quantitative study, patient experiences are limited to the questions in the scales used. Qualitative or mixed studies can be conducted in the future to collect more in-depth data from patients. Secondly, the study results are limited to the general surgery clinic of a university hospital. Finally, the study is further limited by the relatively low frequency of pain and sleep assessments conducted within the first 24 h following MBS, with pain assessed three times and sleep assessed only once. Despite the limitations, the study contributes to the literature by revealing the relationship between the level of surgical fear, early postoperative pain severity, and sleep quality of patients before MBS.

## Conclusion

As a result of the study, it can be said that patients experienced a low level of surgical fear before MBS. It was determined that the pain intensity of the patients was severe in the first postoperative measurement and tended to decrease gradually. Patients had moderate sleep quality on the first postoperative day. In addition, the study found that as patients’ levels of surgical fear increased, their pain intensity also increased, while their sleep quality decreased. It is recommended to consider all factors that may influence pain and sleep quality, including surgical fear, when assessing patients following MBS. In addition, it may be beneficial to develop support and counseling services for patients aimed at reducing surgical fear, based on current guidelines. Furthermore, a collaborative approach among the surgical team (surgeon, nurse, psychologist, etc.) could provide a holistic solution to the pain and sleep issues experienced by patients in the postoperative period. Qualitative studies that provide a deeper understanding of the subjective experiences of surgical fear, pain experience, and sleep quality in this patient group may also be recommended.

## Data Availability

The datasets generated during and/or analysed during the current study are available from the corresponding author on reasonable request.
